# Advanced Nanomedicine Delivery Systems for Cardiovascular Diseases: Viral and Non-Viral Strategies in Targeted Therapy

**DOI:** 10.3390/molecules30040962

**Published:** 2025-02-19

**Authors:** Qian Chen, Tong Yu, Jingyi Gong, Hongli Shan

**Affiliations:** Shanghai Frontiers Science Research Center for Druggability of Cardiovascular Noncoding RNA, Institute for Frontier Medical Technology, College of Chemistry and Chemical Engineering, Shanghai University of Engineering Science, Shanghai 201620, China; m340122502@sues.edu.cn (Q.C.); m340223138@sues.edu.cn (J.G.)

**Keywords:** cardiovascular disease, nanomedicine delivery system, viral vectors, non-viral vectors, targeted therapy

## Abstract

Cardiovascular diseases (CVDs) represent a leading global health crisis, significantly impairing patients’ quality of life and posing substantial risks to their survival. Conventional therapies for CVDs often grapple with challenges such as inadequate targeting precision, suboptimal therapeutic efficacy, and potential adverse side effects. To address these shortcomings, researchers are intensively developing advanced drug delivery systems characterized by high specificity and selectivity, excellent biodegradability, superior biocompatibility, and minimal toxicity. These innovative systems enable the precise delivery of pharmaceuticals with high drug-loading capacities, minimal leakage, and expansive specific surface areas, thereby enhancing therapeutic outcomes. In this review, we summarize and classify various drug delivery materials targeting CVDs and application values. We also evaluate the feasibility and efficacy of viral and non-viral vectors for the treatment of CVDs, the existing limitations and application prospects are also discussed. We hope that this review will provide new perspectives for the future development of drug delivery systems for the treatment of CVDs, ultimately contributing to improved patient care and outcomes.

## 1. Introduction

CVDs represent a significant global health concern, with high rates of morbidity, mortality, and complications. They account for approximately one-third of global deaths, significantly contributing to the global economic burden [1,2]. Current therapeutic strategies can be divided into three categories: (1) pharmacological treatments (e.g., lipid-lowering drugs and antihypertensives), (2) surgical procedures (e.g., coronary artery bypass grafting), and (3) catheter-based interventions utilizing implantable devices such as drug-eluting stents (DESs) and balloons (DEBs) [3,4]. Although DES/DEB dominate percutaneous coronary interventions, the problem of poor long-term prognosis remains. This review specifically excludes all device-mounted systems, focusing on injectable and systemic drug/gene delivery platforms. These advanced systems show unique advantages in precision targeting (e.g., ligand-guided nanoparticles (NPs) [5], bionanoparticles [6], etc.), and pathology-specific adaptations (e.g., stimulus-responsive release in the ischemic microenvironment [7], etc.) offering transformative potential beyond the anatomical limitations of implantable devices.

Conventional pharmacological interventions often produce systemic side effects due to non-specific biodistribution and off-target effects [8]. To overcome these limitations, targeted drug delivery systems (DDSs) have emerged as a promising strategy to improve therapeutic precision. Compared to invasive procedures such as coronary artery bypass grafting, which carry the risk of post-operative complications and high costs [9], DDSs offer a less invasive alternative with an improved safety profile. However, the clinical translation of DDSs faces challenges including complex design requirements, low success rates in preclinical models, and long development timelines [10,11]. Overcoming these hurdles requires innovations in nanomaterial engineering, such as surface functionalization for active targeting, to achieve site-specific therapeutic efficacy and reduce systemic toxicity. It has a wide range of potential applications in the treatment of cancer, cardiovascular disease, and neurological disorders.

Nanomedicine delivery systems have made significant advances in the treatment of cardiovascular disease, particularly in the application of bionanoparticles, the potential of organ-on-a-chip technology, and innovations in multifunctional NPs. Bionic NPs are of interest for their precise delivery and clinical suitability advantages [12]. Organ-on-a-chip technology has been intensively studied for the first time for drug toxicity and efficacy assessment, providing new ideas for the clinical translation of biomimetic and stimuli-responsive nanosystems [13]. Meanwhile, multifunctional NPs targeting specific cardiovascular diseases (e.g., pulmonary hypertension therapy) are emerging, expanding the range of NPs. These studies have also laid the groundwork for breakthrough applications in areas such as COVID-19 mRNA vaccines [14]. This review focuses on targeted delivery systems (DDSs) for cardiovascular diseases, highlighting the design of viral and non-viral vectors and their application in gene therapy. The main strategies include ligand-guided targeting, stimulus-responsive design, and immune cell-mediated delivery. In addition, the review explores the potential and challenges of nanomedicine in the treatment of cardiomyopathy and atherosclerosis, emphasizing the need to overcome limitations in targeting efficiency, immunogenicity, and clinical translation to improve patient prognosis.

## 2. Conventional Delivery Strategies Targeting the Cardiovascular System

The emergence of nanotechnology has led to new approaches in the field of DDSs, which offers hope for the management of CVDs. This novel drug delivery system is designed to enhance the efficacy, safety, and compliance of pharmaceuticals by regulating the delivery and release of drugs, optimizing pharmacokinetic and pharmacodynamic properties, promoting drug absorption across physiological barriers, and increasing drug bioavailability [15]. The physical chemistry properties and surface modifications of NPs can be used to achieve targeted drug delivery to the cardiovascular system [16]. There are two main targeting strategies, including active and passive targeting [17]. The difference between these two strategies is that passive targeting relies on the pathophysiological characteristics of the diseased tissue [18], whereas active targeting achieves superior spatial control of drug delivery by actively guiding the drug carrier to a predetermined biological target via an artificial recognition system [19]. NPs are capable of acting as drug carriers to deliver therapeutic drugs, including small-molecule drugs, proteins, nucleic acids, and cytokines, to pathological sites [20]. The use of NPs as carriers of targeted cardiovascular drugs represents a major advance in the treatment of cardiovascular disease.

### 2.1. Passive Targeted Delivery

Passive targeting usually utilizes physiological structural characteristics to achieve the natural distribution of drug differences in the body [21]. The enhanced permeability and retention (EPR) effect refers to the selective accumulation and long-term retention of macromolecular drugs in tumor tissues due to the high permeability and structural abnormalities of blood vessels around tumor tissues. This provides a theoretical basis for the enrichment of nanomedicines in tumor tissues [22]. In addition to the EPR effect that can be observed in tumor models, the properties of the site of the lesion can be similarly exploited in the cardiovascular field to prolong drug retention for ameliorative and therapeutic purposes [23]. In peripheral arterial disease, the passive accumulation of NPs in ischemic tissue is achieved by the EPR effect, and their accumulation is positively correlated with the degree of ischemia. As blood perfusion improves, the EPR effect diminishes and NP accumulation decreases [24]. However, the EPR effect in the context of cardiovascular disease is considerably less prolonged than in tumors, and may not be sufficient for drug delivery. Furthermore, the integrity and hemodynamic characteristics of the vasculature also restrict the passive targeting of drugs to the lesion site [25]. In atherosclerosis, while there may be inflammation and damage to the vessel wall, the integrity of the vessel prevents the drug from easily passing through the vessel wall, thereby limiting drug penetration and delivery. Similarly, in areas of ischemia, blood flow may be slowed or completely interrupted, which affects the distribution of the nanomedicine and its ability to reach the lesion [26]. Therefore, in the field of cardiovascular disease therapy, although initial drug aggregation in the diseased region can be achieved using the EPR effect, this effect is not sufficient to achieve the desired efficacy. There is an urgent need to develop DDSs capable of precisely targeting damaged cardiovascular tissues to enhance targeting precision and prolong the duration of drug efficacy.

### 2.2. Active Targeted Delivery

Considering the inherent constraints of passive localization, an active localization strategy that can provide better control and accuracy is adopted to achieve precise drug delivery. A receptor or molecular marker that modifies a drug carrier with a ligand or antibody to localize to a specific lesion area is discussed in [27]. Recent studies have shown that the surface modification of NPs can be used to target specific cell types, such as endothelial cells and smooth muscle cells. Using cardiac endothelial-targeting peptide (CRPPR)-modified nanocarriers that can specifically recognize and bind cardiac endothelial cells, therapeutic nucleic acids can be efficiently delivered to the site of myocardial infarction, significantly improving therapeutic efficacy [28]. In the treatment of atherosclerosis, the strategy of targeting smooth muscle cells follows the same principle [29]. Another approach is active targeting based on nanocarrier properties. Active targeting strategies based on nanocarrier properties in cardiovascular therapy mainly include the design of pH-responsive and enzyme-responsive nanocarriers [30]. pH-responsive nanocarriers are able to undergo structural changes in specific pH environments to achieve precise release of the drug, thus improving the therapeutic effect. For example, in atherosclerosis, under the acidic microenvironment at the lesion site, the surface of the carrier responds to the pH change and controls the drug release through the hydrolysis of the Schiff group [31]. Enzyme-responsive nanocarriers, on the other hand, make use of matrix metalloproteinases (MMPs), which are highly expressed specifically in myocardial infarction regions, and when NPs reach myocardial infarction sites, the MMPs will prompt the release of drugs, achieving drug enrichment at the lesion site and effective treatment [32]. In addition, responsive nanocarriers developed for enzymes with altered activity in atherosclerotic plaques can precisely deliver plaque stability-regulating, anti-inflammatory, and lipid-lowering drugs into the plaque to improve its pathological state and reduce the risk of cardiovascular events [33]. In addition, targeted drug delivery can be achieved by exploiting the natural homing ability of immune cells (e.g., macrophages and monocytes) and stem cells [34,35,36]. Immune cells can load drugs and migrate towards the site of the lesion, and in myocardial infarction or inflammatory cardiovascular disease, macrophages congregate to the damaged area, releasing drugs and modulating the inflammatory response and tissue repair [37]. Meanwhile, stem cells such as mesenchymal stem cells act as drug carriers and are able to express therapeutic proteins or secrete factors that promote cardiomyocyte survival and repair, improving cardiac function [38]. In addition, drugs or gene editing tools can also be mounted on stem cells to achieve the precise treatment of diseased areas [39]. Therefore, achieving precise delivery and improving therapeutic efficacy through multiple modalities has great potential in the treatment of cardiovascular diseases.

## 3. Delivery Vehicles Targeting Cardiovascular Disease

**Figure 1:**
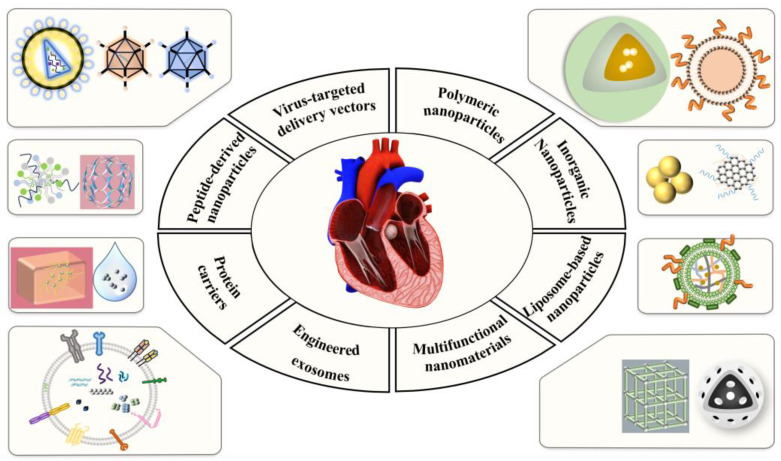
Classification of delivery vectors targeting CVDs. Viral and non-viral vectors are used for cardiovascular drug delivery. Viral vectors include lentiviruses, adenoviruses, and adeno-associated viruses. Non-viral vectors comprise polymeric NPs, inorganic NPs, liposome-based NPs, functional NPs, engineered exosomes, protein carriers, and peptide-derived NPs.

In the field of cardiovascular disease treatment, research on delivery systems is becoming a pivotal factor in driving drug development. As a result of a more profound comprehension of disease mechanisms and the rapid advancement of nanotechnology and biomaterials science, delivery systems demonstrate considerable potential for achieving efficacious, targeted, and secure therapies. Notwithstanding the numerous challenges associated with targeting [40], biocompatibility [41], and immunogenicity [42], the design and optimization of novel delivery systems are gradually overcoming these difficulties. Next, we will focus on the application of several leading-edge DDSs, including viral and non-viral vectors, in the treatment of CVDs (Figure 1). The design strengths, challenges, and future directions of these systems will also be discussed.

### 3.1. Virus-Targeted Delivery Vectors

Gene therapy is a technique for treating specific diseases by modifying an individual’s genes. Since the 1980s, gene therapy has emerged as an innovative medical treatment [43], based on the delivery of healthy or therapeutic genes to target cells through specific vectors, with the aim of repairing or replacing defective genes and thereby treating diseases [44]. In this process, viral vectors have become a commonly utilized tool in the field of gene therapy due to their exceptional delivery efficiency [45]. The most commonly utilized viral vectors for delivery are lentivirus, adenovirus, and adeno-associated virus.

#### 3.1.1. Lentiviral Vectors

Lentiviral (LV) vectors, derived from human immunodeficiency virus, are powerful tools for modifying eukaryotic genes such as hematopoietic stem cells and neural cells [46,47]. They are mainly used for gene therapy and gene function research, especially for cell types that require long-term and stable expression, showing great potential in gene therapy for CVDs [48]. LV vector-mediated gene transfer of viral-derived interleukin-10 (vIL-10) has been used to prolong the survival time of cardiac allografts in a cardiac transplantation model. vIL-10 expression inhibits T-cell proliferation, and thus may have a positive effect on suppressing immune rejection after cardiac transplantation [49]. In addition, LV vectors have been evaluated for gene transfer efficiency and safety in cardiomyocytes, providing important experimental data and therapeutic strategies for gene therapy of CVDs [50].

#### 3.1.2. Adenoviral Vectors

Adenoviral (AdV) vectors were first detected originating from human adenoid tissue and were used as gene delivery vectors in the 1980s [51]. They are distinguished by their capacity to infect both dividing and non-dividing cells, which renders their utilization in non-proliferating tissues, such as the heart, a viable proposition. Although its mediated gene expression is transient, it usually peaks a few days after gene transfer and lasts for 2–4 weeks [52]. In the field of CVDs, AdV is used to deliver therapeutic genes such as vascular endothelial growth factor (VEGF) to promote myocardial neovascularization and improve ischemia. Recently, XC001, a novel AdV vector that expresses multiple VEGF isoforms, has been shown to safely and effectively promote angiogenesis. The EXACT phase II trial evaluated the safety and preliminary efficacy of XC001 in patients with refractory angina pectoris and demonstrated that it was safe to treat and had a positive effect in terms of improvement in exercise capacity, myocardial perfusion, and angina symptoms [53]. These studies support the potential and promise of AdV vectors in gene therapy for cardiovascular disease.

#### 3.1.3. Adeno-Associated Viral Vectors

Adeno-associated viral (AAV) vectors are small, single-stranded DNA viruses that have received much attention for their potential in gene therapy. The delivery efficiency and specificity of AAV vectors are its key advantages in the treatment of CVDs. Studies have shown that AAV vectors can efficiently transduce cardiac tissues, especially cardiomyocytes and cardiac fibroblasts, through precise serotype selection and targeting strategies. For example, AAV9 has been extensively studied for its ability to cross the blood–brain barrier and efficiently transduce multiple cell types [54]. Using AAV9 as a vector carrying specific genes driven by a modified osteopontin promoter, it is designed to precisely deliver therapeutic genes to specific cell populations in damaged areas of the heart. The expression of these genes significantly contributed to the regeneration and repair mechanisms of cardiomyocytes [55]. In addition, AAV vectors have also been used in the study and treatment of myocardial hypertrophy. AAV specifically inhibits the expression of genes that promote cardiac hypertrophy, such as miR-23a and miR-182, through gene editing technology or gene silencing strategies, which in turn reduces myocardial fibrosis and protects the structure and function of the heart [56,57]. The use of AAV vectors in cardiovascular disease is focused on the treatment of myocardial infarction, myocardial hypertrophy, and atherosclerosis [58,59,60]. AAV-mediated gene delivery enables the targeting of specific cell types to improve disease progression and patient prognosis [61].

Each of the three viral vectors is appropriate for distinct types of cardiovascular disease therapy. The characteristics of all the viral vectors described above are summarized in Table 1. Future research must prioritize the enhancement of vector safety and efficacy while minimizing potential adverse effects to fully harness the potential of these viral vectors in the treatment of CVDs.

### 3.2. Non-Viral Targeted Delivery Vectors

Non-viral vectors are a class of vectors used for gene delivery that do not involve viruses. They are less immunogenic and safer because they do not use live viral components [62]. Non-viral vectors, as a type of nanodrug carriers, take advantage of their nanoscale properties to encapsulate, disperse, or non-covalently or covalently bind drugs to nanocarriers through specific preparation processes for precise drug delivery. These non-viral carriers (Table 2), including liposome-based NPs, polymeric NPs, inorganic NPs, and engineered exosomes, are selected according to the needs of a specific disease in terms of the body material and surface modification for drug delivery, and the ideal nanoparticle formulations should have the following characteristics: biocompatible, biodegradable, optimal particle size, surface charge, modifiable surfaces, targeting, and long blood circulation time [63].

#### 3.2.1. Liposome-Based Nanoparticles

**Figure 2:**
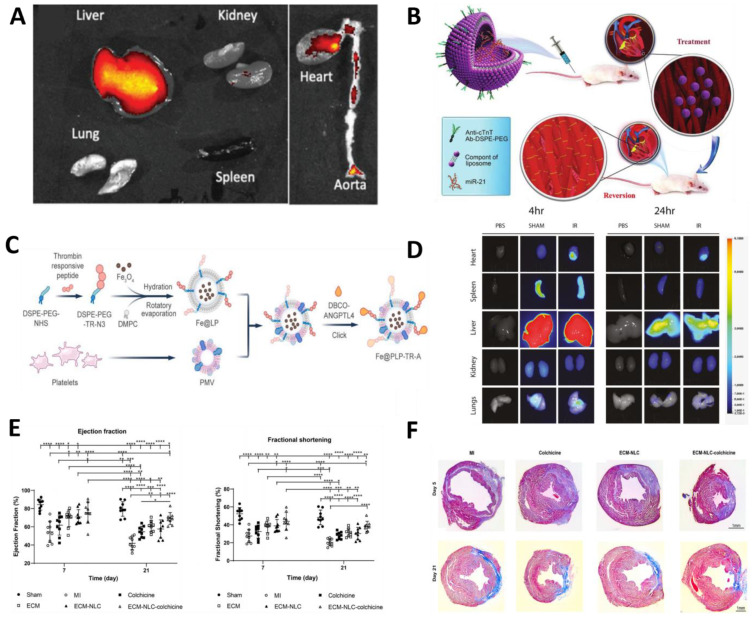
Modified liposome-based NPs targeting CVDs. (**A**) Representative near-infrared fluorescence (NIFR) images of liposome accumulation in various organs at 24 h post-injection. Reproduced with permission from Ref. [71]. Copyright: *Adv. Healthc. Mater*. 2020. (**B**) Schematic of cT-21-LIP targeting cTnT for AMI. Reproduced with permission from Ref. [72]. Copyright: *J. Mater. Chem. B* 2020. (**C**) Schematic of Fe@PLP-TR-A preparation. Reproduced with permission from Ref. [73]. Copyright: *Chem. Eng. J.* 2022. (**D**) Biodistribution of fluorescently labeled LNP measured by whole-organ fluorescence spectroscopy. The colour bars indicate a range of values for signal strength from 3.72 × 10^−3^ to 6.1 × 10^0^.Reproduced with permission from Ref. [74]. Copyright: *J. Control. Release* 2022. (**E**) Increased ejection fraction (EF) and Fractional Shortening (FS) in the ECM-NLC-colchicine group compared with colchicine treatment on days 7 and 21 after myocardial infarction. * *p* < 0.05, ** *p* < 0.01, *** *p* < 0.001, **** *p* < 0.0001. (**F**) Detection of cardiac fibrosis after myocardial infarction by MT staining after days 5 and 21. Reproduced with permission from Ref. [75]. Copyright: *Biomaterials* 2023.

Liposome-based NPs comprise a variety of substructures, most typically a spherical platform containing at least one lipid bilayer internally, typically divided into liposomes and lipid nanoparticles (LNPs) [64]. The basic structure of liposomes is the lipid bilayer, which resembles the bilayer of the cell membrane and contains different types of phospholipids, including cationic, anionic, or neutral lipids and cholesterol, which collectively surround the water a aqueous nucleus. This distinctive structure enables liposomes to transport hydrophobic compounds through their lipid bilayer while carrying hydrophilic compounds in their internal aqueous core, thereby facilitating the efficient encapsulation and delivery of drugs [65,66]. To improve the stability of liposomes in vivo and extend their cycle time, the researchers achieved this by modifying a layer of the hydrophilic polymer polyethylene glycol on the surface of the liposomes [67]. The polyethylene glycol (PEG) coating not only enhances the ability of liposomes to resist enzymatic degradation and removal in vivo, but also reduces non-specific interactions with plasma proteins, thereby improving liposome stability and drug bioavailability [68,69]. Berberine is an alkaloid with anti-inflammatory and cardioprotective effects, but its poor aqueous solubility and short half-life limit its application. Researchers used PEG-modified long-circulating liposomes to improve its bioavailability, demonstrating IL-6 secretion inhibition in RAW 264.7 macrophages in vitro and cardiac function preservation in a C57BL/6J mouse myocardial infarction model in vivo, targeting drug delivery to inflamed cardiac tissues through a passive concentration effect, enhancing efficacy and reducing systemic distribution [70]. In the production of arteriosclerosis foam cells, fluocinolone acetonide is an anti-inflammatory corticosteroid that reduces lipid accumulation and inflammatory cytokines. To directly target atherosclerotic plaques and reduce systemic exposure, an injectable flupropiophenone acetonide nanoliposome was developed for sustained release over 25 days to promote cholesterol efflux, demonstrating anti-inflammatory effects and enhanced cholesterol efflux in THP-1-derived foam cells in vitro and plaque accumulation with reduced macrophage content in apolipoprotein E-knockout (Apoe^−/−^) mouse atherosclerotic models in vivo. The PEG-modified liposomes remain stable during circulation, preventing premature drug leakage and ensuring sustained release and action at the plaque site (Figure 2A) [71]. These studies indicate that PEG-modified liposomes have important application value and development prospects in drug delivery for cardiovascular diseases.

Moreover, ligand-targeted liposomes have been developed with the objective of achieving precise drug delivery. The attachment of specific ligands, such as antibodies, short peptides, and saccharides, to the surface of the liposome or to the end of the PEG chain enables the targeting of specific cells or tissues. The ligand modification enables liposomes to specifically bind to cell surface receptors, thereby promoting cellular uptake and efficient drug delivery [76]. This targeting strategy enables the drug to be more precisely focused on the diseased area, thereby reducing damage to normal tissues and improving medical efficacy. For example, cTnT antibody-modified liposomes can specifically bind cTnT on the surface of cardiomyocytes in vitro (hypoxia-treated primary cardiomyocytes) and in vivo (AMI rat models) to deliver miR-21 mimics to improve cardiac function and reduce infarct size in the treatment of acute myocardial infarction (AMI) (Figure 2B), showing better targeting ability than PEG-modified liposomes [72]. In MI/RI, platelet membrane and thrombin-responsive peptide-modified liposome systems in vitro (hypoxia-treated HUVECs) and in vivo (mouse myocardial ischemia–reperfusion models) stabilize vascular endothelial junctions, reduce vascular gaps, and effectively improve cardiac function (Figure 2C). In addition, this system utilizes a thrombin-responsive peptide that responsively releases the drug in an environment of locally high concentrations of thrombin, resulting in microenvironment-responsive release. This study further highlights the advantages of ligand-modified liposomes over PEG modification [73]. PEG modification mainly focuses on improving the overall stability and circulation time of liposomes, with relatively weak target recognition of specific cells; whereas ligand-modified liposomes have significant advantages in precision drug delivery, and can precisely intervene in the physiological processes of diseased cells during the disease process, providing an effective strategy for cardiovascular disease treatment.

LNPs are an advanced class of liposome-based nanocarriers for efficient delivery of nucleic acid drugs, with cores consisting of cations or ionizable lipids that interact with nucleic acids via electrostatic interactions and facilitate their release from the endosomal environment. Phospholipids and cholesterol contribute to the structural integrity and stability of the particles. Phospholipids and cholesterol contribute to improving the structural integrity and stability of particles. Furthermore, the incorporation of polyethylene glycolated lipids prolongs the circulation time of LNPs in the blood, which demonstrates the potential of this nanomaterial in the development of tailored gene therapy strategies [77]. Specifically, ionizable LNPs effectively promote the intracellular delivery of nucleic acids due to their neutral properties under physiological conditions and their ability to undergo charge conversion in an acidic environment.

Modified mRNA (modRNA) is emerging as a promising approach for the treatment of CVDs due to its enhanced stability and reduced immunogenicity. Liposomal nanoparticles (LNPs) act as a delivery system to efficiently deliver modRNAs to damaged myocardial tissues (Figure 2D), promoting cardiac repair and regeneration. In vivo studies using murine models of myocardial ischemia–reperfusion injury demonstrated that intravenously administered LNPs accumulated preferentially in the infarcted myocardium, leveraging vascular permeability induced by ischemic damage. This strategy is highly promising for treating myocardial ischemia–reperfusion injury. Compared with conventional naked modRNA, which was tested in direct intramyocardial injection models but showed susceptibility to nuclease degradation and poor targeting efficacy, LNPs enabled systemic administration with enhanced lesion-specific delivery. Functional validation in Cre-reporter mice further confirmed the cell-type-specific uptake of LNP-encapsulated modRNA, predominantly targeting cardiac fibroblasts, cardiomyocytes, and macrophages within the infarct region. These preclinical findings highlight the translational potential of LNPs for cardiac regeneration therapies [74]. From passive to active targeting, the use of LNPs demonstrates flexibility and effectiveness in the treatment of cardiovascular disease. In another study, the researchers employed the ligand modification approach, in which they developed CD5-modified LNPs, which confer the ability to specifically recognize and bind to fibroblast-activating protein (FAP) on the surface of activated cardiac fibroblasts, enabling precise active targeted delivery. In a mouse model of heart failure, this strategy effectively generated CAR T cells, reduced fibrosis, and significantly improved cardiac function. Specific effects were demonstrated by a reduction in ventricular volume, improvement in diastolic and systolic cardiac function, and a reduction in collagen deposition [78]. Another approach is in situ injection, which reduces the systemic distribution of the drug and decreases systemic side effects compared to traditional systemic administration. To achieve this strategy, the researchers cross-linked nanostructured lipid carriers (NLCs) with cardiac extracellular matrix (ECM) fibronectin to form an ECM-NLC hydrogel. The ECM-NLC hydrogel, carrying colchicine, which has anti-inflammatory effects, was injected locally at the site of cardiac injury, and the drug was released continuously over a 2-week period to improve cardiac function and reduce fibrotic effects (Figure 2E,F) [75]. These studies have demonstrated promising therapeutic effects in specific disease models and have significant developmental implications in the cardiovascular field.

#### 3.2.2. Polymer Nanoparticles

Polymer NPs are a class of NPs ranging 1–1000 nm in size and are composed of a variety of polymer components, which exhibit a high degree of flexibility in design concepts [79]. Active compounds are encapsulated in the polymer core or adsorbed on the surface by chemical coupling with NPs. Polymer NPs offer three principal advantages in the field of drug delivery: adjustable size, customizable shape, and tunable surface charge [80]. These properties enable the accommodation of a variety of therapeutic drugs, including hydrophobic and hydrophilic compounds, as well as biologically active substances with disparate molecular weights, such as small molecules, biomolecules, proteins, and vaccines [81,82]. This renders them an optimal vehicle for the co-delivery of a diverse range of payloads, thereby providing a formidable instrument for personalized medicine and precision therapy. Polymer NPs are typically classified into two main categories: natural polymers and synthetic polymers.

**Figure 3:**
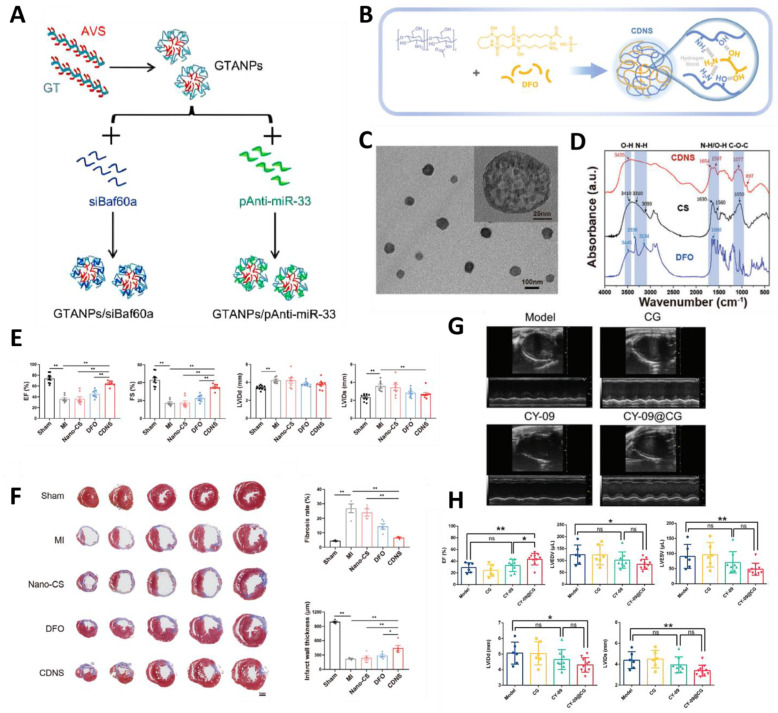
Natural polymer targeting CVDs. (**A**) Preparation of GTANPs/siBaf60a and GTANPs/pAnti-miR-33. Reproduced with permission from Ref. [87]. Copyright: *Biomaterials* 2022. (**B**) Schematic of the binding model of chitosan (CS) and deferoxamine (DFO). (**C**) Cryo-transmission electron microscopy (cryo-TEM) image of CDNS. (**D**) Fourier transform infrared spectra (FT-IR) spectra of CS, DFO, and CDNS. (**E**) Quantification of the EF, FS, LVIDd, and LVIDs of the hearts of different groups at 28 days after MI. (**F**) Representative Masson trichrome staining and quantification of fibrotic areas and infarct wall thickness of the hearts of different groups at 28 days after MI. images. * *p* < 0.05, ** *p* < 0.01. Reproduced with permission from Ref. [88]. Copyright: *Adv. Sci*. 2024. (**G**,**H**) Ultrasound images of CY-09@CG at 5 weeks after improvement of I/R cardiac injury. and corresponding quantitative assessment of EF, LVEDV, LVESV, LVIDd, and LVIDs. * *p* < 0.05, ** *p* < 0.01, ns: no significant difference. Reproduced with permission from Ref. [89]. Copyright: *Adv. Funct. Mater.* 2022.

Natural polymers have good biocompatibility and biodegradability and can be chemically modified to enhance their functionality. They can bind negatively charged drugs through electrostatic interaction for targeted delivery and play a synergistic role in the treatment of cardiovascular diseases, improving drug efficacy and cardiac function. Chitosan and dextran, as classical natural polymer NPs, play an important role in the treatment of CVD. Chitosan enhances drug biodistribution, reduces pharmacological toxicity, and improves specificity and sensitivity [83]. However, due to its low mechanical strength and susceptibility to degradation, chitosan often needs to be chemically modified, cross-linked, or complexed with other materials to meet specific needs. For example, different functional groups are introduced through chemical reactions such as acylation, carboxymethylation, and phosphorylation, to enhance its solubility, antimicrobial properties, and other specific functions. The positive charge and bioadhesive properties of chitosan allow it to bind to negatively charged nucleic acids by electrostatic interaction for nucleic acid drug delivery [84,85,86]. Common chitosan NPs may be loaded with drugs only by simple physical adsorption or encapsulation, which may lead to the poor stability of the loaded drug, which has been improved by the researchers in atherosclerosis treatment. Firstly, galactose-modified trimethyl chitosan nanoparticles (GTANPs) were synthesized, and then coupled with hydrophobic atorvastatin via amide/ester bonding, followed by the electrostatic encapsulation of Baf60a siRNA and anti-miR-33 pDNA (pAnti-miR-33) (Figure 3A); NPs delivering statins and nucleic acids were prepared for the treatment of atherosclerosis. The in vitro and in vivo experiments showed that these NPs synergistically reduced plasma cholesterol and low-density lipoprotein cholesterol levels, significantly reduced plaque area, and inhibited the accumulation of inflammatory cells in plaques [87]. In addition to its application in atherosclerosis, researchers have also prepared different modifications of chitosan based on different mechanisms of occurrence in myocardial infarction. Myocardial infarction leads to the degradation of ferritin and the production of excess free iron, leading to ferroptosis. The covalent cross-linking of chitosan with desferrioxamine results in the formation of nano-sponges (CDNSs) with a spongy and porous structure (Figure 3B–D), which gives them a larger specific surface area and allows for the direct chelation of ferric ions without the need for releasing desferrioxamine. The structure of the CDNS can be efficiently absorbed by cardiac myocytes with good biocompatibility, which achieves intracellular ion chelation, reduces oxidative stress, promotes angiogenesis, improves cardiac function, and reduces cardiac remodeling. In in vivo experiments in a mouse model of myocardial infarction, CDNS exploited the microenvironmental characteristics of the local ischemic environment after myocardial infarction, especially the change in pH, to achieve the responsive release of the drug, and showed significant therapeutic effects, including the inhibition of iron death and the improvement of cardiac function. (Figure 3E,F) [88]. Dextran is another biocompatibility natural polysaccharide that can be recognized by immune cells through specific receptors (e.g., dectin-1), which act as targeted ligands to deliver drugs directly to specific cells or tissues, improve drug efficacy, and reduce the damage to normal cells. Studies have shown that dectin-1^+^ monocytes/macrophages are the major and specific mediators of MI/RI. On this basis, the researchers prepared β-glucan NPs containing the small-molecule inflammasome inhibitor Cy-09, which enhanced MI/RI region accumulation and increased the efficacy of Cy-09, reduced MI/RI-induced myocardial injury, and improved cardiac function (Figure 3G,H). These findings were validated both in in vitro models using macrophages and in in vivo models using mice with surgically induced myocardial infarction [89].

**Figure 4:**
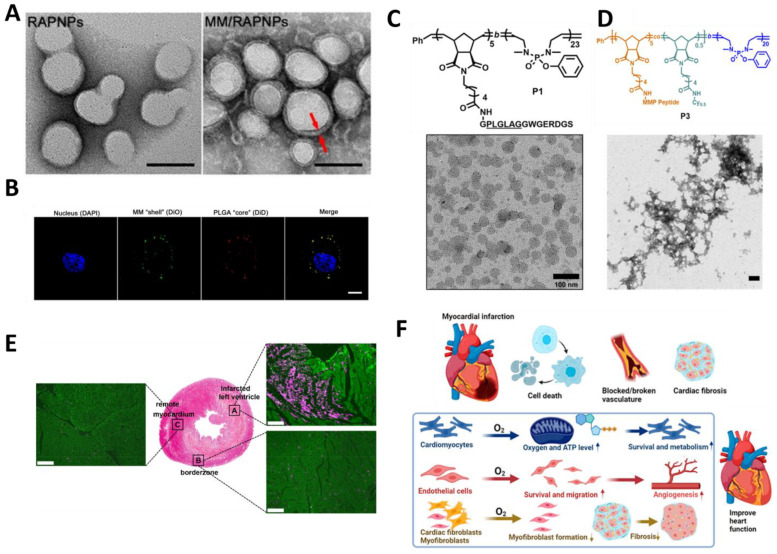
Synthetic polymers targeting CVDs. (**A**) TEM images of RAPNPs and MM/RAPNPs. (**B**) CLSM images of the colocalization of the nucleus (blue), MM “shell” (green) and PLGA “core” (red). Reproduced with permission from Ref. [91]. Copyright: *Theranostics* 2021. (**C**) TEM image of P1 assembled into spherical micelles. (**D**) TEM image of P3 with S. thermophilus protease at 1:100 S. thermophilus protease: polymer in DPBS incubated at 37 °C for 24 h to form aggregates. (**E**) Hematoxylin–eosin (he) stained heart sections. α-actinin staining (green), Cy5.5-labeled degradable micellar nano-particles (DNPs) are shown in red. Reproduced with permission from Ref. [26]. Copyright: *ACS Nano* 2022. (**F**) Mechanism of oxygen-mediated cardiac repair and heart function restoration. Reproduced with permission from Ref. [92]. Copyright: *J. Am. Chem. Soc.* 2023.

Synthetic polymer NPs are prepared by chemical or physical methods at the nanoscale and possess unique properties and application potential due to their particle structure, including a high specific surface area, quantum size effect, easy biodegradability, and adjustability. In the treatment of CVD, synthetic polymer NPs can achieve targeted drug delivery and controlled release, improve efficacy and reduce adverse reactions by encapsulating drugs in NPs [90]. To improve the targeting and biocompatibility of NPs in vivo, based on the fact that macrophages play an important role in plaque formation in the early stages of atherosclerosis, the researchers developed a biomimetic NPs based on macrophage membrane functionalization (MM/RAPNPs) (Figure 4A,B) loaded with rapamycin for targeted delivery to the lesion site. In vitro experiments evaluating the interaction of MM/RAPNPs with macrophages demonstrated their potential for targeted delivery. In addition, animal model studies have shown that MM/RAPNPs can accumulate within atherosclerotic plaques after the intravenous injection of MM/RAPNPs. This accumulation was mainly achieved through EPR effects in atherosclerotic lesions, which promoted the removal of necrotic and inflammatory mediators from plaques and reduced plaque growth [91]. Extracellular matrix metalloproteinases (MMPs) are upregulated after AMI. Researchers have prepared novel degradable phosphate NPs containing a specific norbornene monomer and peptide chain. The MMPs recognizes and cleave this specific peptide chain, making the NPs targetable in myocardial infarction models. These NPs transformed from a micellar state to micron-sized aggregates in the presence of MMPs (Figure 4C,D), contributing to the retention and local enrichment of the drug in the region of injury (Figure 4E) By in vitro assay, the NPs are responsive to MMPs and do not cause significant cellular changes in metabolic activity and hemolysis time. Moreover, in a rat model of MI, its accumulation in the infarcted heart was significantly increased, and it was cleared predominantly by the kidneys [26]. The design of such NPs focuses on their biodegradability, as well as their responsiveness to the inflammatory environment, allowing them to accumulate and be cleared on relevant time scales. In addition, rapid continuous oxygenation after AMI is an effective method to protect myocardial cells and restore cardiac function. However, existing oxygen-producing materials can only be used during AMI by direct injection or suturing, both of which carry the risk of triggering rupture of cardiac tissue. To address this challenge, Y. Liang et al. designed oxygen-releasing NPs with a biodegradable polymer shell and core containing a polyvinylpyrrolidone (PVP)/hydrogen peroxide (H_2_O_2_) complex. The administration of these NPs resulted in a significant enhancement in the survival of cardiomyocytes, cardiac fibroblasts, and endothelial cells under hypoxic conditions. Following intravenous injection during AMI, the NPs were able to target the infarcted heart and specifically accumulate to improve cardiomyocyte survival and stimulate angiogenesis, thus inhibiting fibrosis without triggering severe inflammation and excess reactive oxygen species due to the local inflammatory microenvironment (Figure 4F) [92]. In summary, these NPs are designed to target different pathological mechanisms of cardiovascular diseases and demonstrate diverse therapeutic strategies, which provide valuable ideas and references in the development of nanotechnology for the personalized and precise treatment of cardiovascular diseases.

#### 3.2.3. Inorganic Nanoparticles

**Figure 5:**
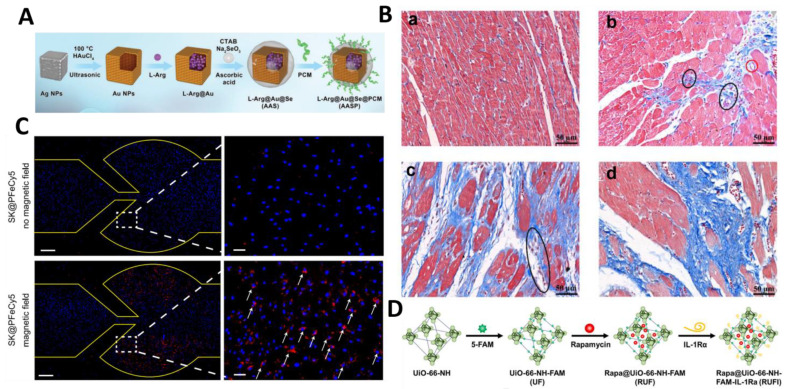
Inorganic NPs targeting CVDs. (**A**) Schematic diagram of AASP synthesis route. Reproduced with permission from Ref. [96]. Copyright: *Adv. Sci.* 2023. (**B**) a: Masson trichrome staining of control heart. b–d: Masson trichrome staining of different locations of MI heart (blood vessels are shown in red circles. Purple spots in black circles represent macrophages). Reproduced with permission from Ref. [97]. Copyright: J. *Control. Release* 2023. (**C**) Fluorescence images of SK@PFeCy5 cell uptake during flow in aortic valve molds with and without magnetic field. The regions pointed by arrows indicate the enriched regions of nanoparticles (SK@PFeCy5) uptaken by cells under magnetic field guidance. Red: SK@PFeCy5; blue: cell nucleus. Reproduced with permission from Ref. [98]. Copyright: *Nat. Commun.* 2024. (**D**) Schematic of the Rapa@UIO-66-NH-FAM-IL-1Ra (RUFI) preparation process. Reproduced with permission from Ref. [99]. Copyright: *J. Control. Release* 2023.

Inorganic NPs can be surface-modified and can bind to drug molecules in different ways, such as electrostatic interactions, hydrophobic interactions, and covalent bonding of groups [93]. Here are several typical inorganic NPs: gold NPs (AuNPs), magnetic NPs (MNPs), metal–organic frameworks (MOFs), and mesoporous silica (MSNs). AuNPs can be modified by various interactions to form a wide range of structures, effectively encapsulate and release drug molecules, and play a key role in drug and gene delivery [94]. In order to improve the safety of AuNPs in cardiac application, the PEG modification of AuNPs was used to inhibit the over-hypertrophy of cardiomyocytes [95]. On the basis of PEG modification, a myocardial-targeting peptide (PCM) was further introduced to modify AuNPs, such as selenium-coated PCM-modified gold nanocages. This modification significantly enhanced the cellular uptake of cardiomyocytes and improved their survival under oxygen glucose deprivation/reoxygenation (OGD/R) conditions. AuNPs could also specifically target infarcted myocardium and significantly improve myocardial function by maintaining mitochondrial function and regulating the NO signaling pathway (Figure 5A). In the treatment of MI/RI, guided by PCM, AuNPs can deliver L-arginine to damaged cardiomyocytes more efficiently, thus improving the therapeutic effect and promoting the recovery of myocardial function [96]. AuNPs with amphiphilic ligands and glucose molecules have been designed to enhance the macrophage uptake and precision of drug delivery, and this modification has a better balance of hydrophilicity and hydrophobicity that facilitates interactions with cell membranes (Figure 5B), thus enhancing their uptake by macrophages and improving the targeting efficiency of the drugs in affected myocardial tissues. In addition, the design achieves microenvironment-responsive release via the enzymatic hydrolysis of glucose in macrophage lysosomes, ensuring that only the amphipathic ligand remains on the surface of the leaked NPs and cannot be re-uptaken by other endogenous cells, thus significantly improving the precision of imaging and targeted delivery and further optimizing its therapeutic efficacy in cardiovascular diseases [97]. MNPs are another important class of inorganic NPs, being mainly used in cancer thermotherapy, and have been used as a novel delivery material during cardiac ischemia–reperfusion injury in recent years. Researchers have developed Fe_3_O_4_-based NPs with a silicon dioxide and PEG modification that can aggregate in damaged cardiac tissue in the presence of a magnetic field (Figure 5C), triggering drug release through the lysosomal acidic microenvironment and the precise release of exosomes to improve cardiac function. In calcific aortic valve disease, MNPs loaded with the lipid-soluble drug XCT790 were used to target and regulate metabolic reprogramming of valve mesenchymal stromal cells, inhibiting osteoblast differentiation and calcium deposition. The nanosystem is dependent on the lysosomal acidic environment for drug-responsive release following PAR2-mediated endocytosis, thereby enhancing therapeutic efficacy [98]. MOFs, which are porous hybrid crystals composed of metal groups and organic ligands, can mimic the structure and function of natural enzymes, possess active sites with multiple catalytic centers, and have been used in drug co-delivery systems. In addition, MOFs can be further modified on their surfaces to increase their functionality. Xu et al. constructed MOF-based NPs, Rapa@UiO-66-NH-FAM-IL-1Ra (RUFI) (Figure 5D), for the treatment of atherosclerotic CVDs. This versatile platform combines drug delivery with immunomodulation and cellular targeting for effective therapy. Specifically, the in vitro effects of RUFI were evaluated using LPS-pretreated RAW264.7 cells, which are murine macrophages, to assess its cytotoxicity and immunomodulatory effects. In vivo, the efficacy of RUFI was tested in an atherosclerotic model of diet-fed ApoE^−/−^ mice, where it demonstrated significant targeting and reduction in atherosclerosis plaques in coronary arteries, carotid arteries, and aortas [99]. With their large surface area and high loading capacity, MSNs are also excellent drug carriers. Studies have shown that MSNs loaded with Notoginsenoside R1 (NGR1) and conjugated with CD11b antibody can be targeted for delivery to the heart, improving cardiac function and modulating macrophage phenotype, inflammatory factors, and chemokines in myocardial infarction mice. Meanwhile, in vitro protective effects of NGR1 against oxidative stress were confirmed using H9C2 cells and primary cardiomyocytes [100]. These studies have shown that different inorganic NPs can play a synergistic role in cardiovascular disease therapy through specific surface modification and functionalization for precise drug delivery, immunomodulation, and cell targeting.

### 3.3. Engineered Exosomes

**Figure 6:**
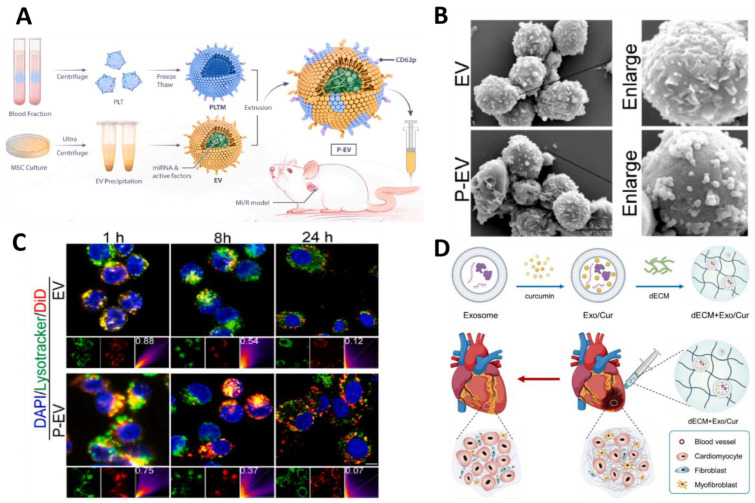
Engineered exosome-targeted therapy for CVDs. (**A**) Schematic of the preparation of P-EV. (**B**) Representative SEM images of THP-1 monocytes after incubation with EV or P-EV for 30 min. (**C**) Representative confocal images of cellular uptake of DiD-labeled EV or P-EV (red) in M1 macrophages stained with lysosomes (green: lysosomes). Yellow color represents co-localization of fluorescent signals from EV and lysosomes. Reproduced with permission from Ref. [104]. Copyright: *Biomaterials* 2022. (**D**) Schematic representation of the preparation of dECM + Exo/Cur and its action. Reproduced with permission from Ref. [105]. Copyright: *J. Mater. Sci. Technol.* 2023.

Engineered exosome is a kind of nano-carrier which can modify exosome by genetic engineering or chemical method to realize a specific function or enhance its performance. Exosomes are endogenous cell-derived NPs, typically between 40 and 100 nm in size, involved in intercellular material transport. They can be released to the outside of the cell through the exogenic effect, mediate cell-to-cell communication, and transfer proteins, mRNA, miRNA, lipids, other substances. Therefore, they can be used as excellent biomimetic nano-carriers for biomedical applications [101]. In recent years, exosomes have emerged as a promising cell-free therapeutic strategy for the treatment of diseases such as AMI, hypertension, heart failure, and cardiomyopathy. At present, exosomes are now entering the clinical trial phase, several of which are registered for the diagnosis and treatment of cardiovascular diseases [102,103]. In the context of immunomodulatory therapies for MI/RI, extracellular vesicles derived from mesenchymal stem cells (MSC-EVs) have demonstrated potential in reprogramming macrophages for immunomodulation. However, their efficacy is limited by poor in vivo targeting. To address this, Li et al. developed platelet membrane-modified EVs (P-EVs) (Figure 6A) that mimic platelet–monocyte binding (Figure 6B), enhancing targeting to ischemic myocardium. In vitro studies demonstrated that P-EVs could bind to monocytes and be efficiently taken up by monocyte-derived macrophages, escaping lysosomal degradation (Figure 6C) and releasing miRNAs that reprogram inflammatory macrophages (M1) to reparative macrophages (M2). In vivo studies confirmed that P-EVs were effectively delivered to the ischemic myocardium, where they were taken up by monocyte-derived macrophages, promoting cardiac repair [104]. Building on this, Wang et al. utilized exosomes encapsulating curcumin, which has antifibrotic properties, and delivered them using a decellularized porcine cardiac extracellular matrix (dECM) hydrogel. This hydrogel provides mechanical support and structural protection, and enhances the water solubility and bioavailability of the exosomes (Figure 6D). In vitro studies demonstrated that these exosomes effectively inhibited the transformation of fibroblasts to myofibroblasts, thereby preventing the progression of fibrosis. The targeted delivery of these exosomes to myocardial infarction sites has been shown to reduce collagen deposition, fibrosis, and infarct size, while improving cardiac function [105]. Engineered exosomes improve targeting and efficacy through surface modification and drug encapsulation in the treatment of cardiovascular diseases, but face challenges such as large-scale production, quality control, and long-term safety, and further research is needed to promote their clinical application.

**Table 2:** Non-viral vector targeted therapy for cardiovascular disease.

Category	Types	Advantages	Model	Applications	Contents	Biological Functions	Refs.
Liposome	BB-lip	Passive	In vitro and in vivo (LAD)	MI	Berberine	Improved ejection fraction and reduced adverse remodeling	[70]
	FA-liposomes	Passive	In vitro and in vivo (Apoe^−/−^ mice)	Atherosclerosis (AS)	Fluoroketone Acetonide (FA)	Anti-inflammatory, promotes cholesterol efflux	[71]
	cT-21-LIPs	Active targeting of cTnT	In vitro and in vivo (LAD)	Acute myocardial infarction (AMI)	miR-21	Reduces apoptosis and infarct size	[72]
	Fe@PLP-TR-A	Active targeting of thrombin peptides	In vitro and in vivo (LAD for 30 min)	MI/RI	ANGPTL4 and Fe_3_O_4_	ROS scavenging effect and protection of endothelial cells from apoptosis	[73]
LNPs	LNPs	Passive	In vitro and in vivo (LAD for 60 min)	MI/RI	modRNA	Reprogramming to reduce fibrosis and promote cardiac repair	[74]
	CD5/LNP-FAPCAR	Active targeting of the T-cell surface protein CD5	In vitro and in vivo (Angiotensin II and Phenylephrine for a week)	Myocardial fibrosis	mRNA	Reduces vascular gap and attenuates fibrosis	[78]
	ECM-NPs	Active (In situ injection)	In vitro and in vivo (LAD)	MI	Colchicine	Improved cardiac function and reduced fibrosis	[75]
Natural Polymer NPs	GTANPs	Active targeting, galactose modification	In vitro and in vivo (ApoE KO mice)	AS	Atorvastatin (AVS) and siBaf60a and pAnti-miR-33	Reduces plasma cholesterol and inhibits plaque formation	[87]
	CDNS	Active targeting, chelates iron ions	In vitro and in vivo (LAD)	MI	Desferrioxamine	Reduced oxidative stress and promoted angiogenesis.	[88]
	CY-09@CG	Active targeting of Dectin-1	In vitro and in vivo (LAD for 30 min)	MI/RI	CY-09	Suppressing Inflammation	[89]
Synthetic polymer NPs	MM/RAPNPs	Active (Macrophage membrane modification)	In vitro and in vivo (Apoe ^−/−^ mice)	AS	Rapamycin	Inhibition of macrophage and smooth muscle cell proliferation and reduction in plaque growth	[91]
	MePTDO	Responding to matrix metalloproteinases	In vitro and in vivo (LAD)	MI	Phosphate NPs	Improve inflammation	[26]
	PCNP/O2	Active (Platelet membrane modification)	In vitro and in vivo (LAD)	AMI	Oxygen-releasing NPs	Promotes angiogenesis and inhibits fibrosis	[92]
Inorganic NPs	PEG-AuNPs	Active targeting of β-adrenergic receptors	In vivo (Isoprotereno-induced myocardial hypertrophy)	Myocardial hypertrophy	/	Reduces cardiac hypertrophy and inflammation	[95]
	AASP	Active (Cardiomyocyte-targeted peptide modification)	In vitro and in vivo (LAD for 30 min)	MI/RI	L-Arginine	Maintains mitochondrial function and inhibits fibrosis	[96]
Inorganic NPs	AuNPs-zwit-glucose	GLUT-1 transporter protein	In vitro and in vivo (Isoprotereno-induced MI)	MI	/	/	[97]
	SR@PFeXCT	Active targeting of protease-activated receptor 2	In vitro and in vivo (①Ldlr ^−/−^ mice; ②Wire injury)	Calcific Aortic Valve Disease	XCT790	Inhibits osteogenic differentiation of VICs and inhibits calcium deposition	[98]
	Rapa@UiO-66-NH-FAM-IL-1Ra (RUFI)	Active targeting Interleukin-1 receptor	In vitro and in vivo (Apoe ^−/−^ mice and carotid artery ligation or carotid collar placement)	AS	Rapamycin and IL-1Ra	Immunomodulation, Cellular Targeting	[99]
	MSN-NGR1-CD11b	Active targeting of macrophage surface antibodies	In vitro and in vivo (LAD)	MI	Notoginsenoside R1 (NGR1)	Promotes angiogenesis and regulates macrophage phenotype	[100]
Engineered exosomes	P-EVs	Active targeting of monocytes	In vitro and in vivo (LAD for 45 min)	MI/RI	miRNA	Regulation of macrophage phenotype, immunomodulation	[104]
	dECM + Exo/Cur	Passive	In vitro and in vivo (LAD)	MI	curcumin	Reduces collagen deposition, fibrosis, and infarct size	[105]

## 4. Conclusions and Perspective

The treatment of CVDs is confronted with a number of challenges, including limitations in remedial efficacy, management of side effects, and specificity to target tissues. With the advent of nanotechnology, novel DDSs, including liposome-based NPs, polymeric NPs, inorganic NPs, and exosomes, present promising strategies for the treatment of CVDs. These systems can enhance drug bioavailability, mitigate adverse effects, and optimize therapeutic efficacy. Liposomes and LNPs, as a model of a DDS, have shown potential in CVD treatment by encapsulating drugs of different properties and by enhancing circularity and targeting through modifications like PEGylation [106]. Polymeric NPs provide a customizable platform for personalized medicine, while inorganic NPs like Au and magnetic NPs, MOFs, and MSNs provide unique properties for CVD therapy [107,108]. Engineered exosomes with their biocompatibility and low immunogenicity are expected to be used in the treatment of CVD, improving precision and efficacy while reducing damage to healthy tissues [109]. Although these nano-delivery systems have shown great potential in experimental studies, their clinical application still faces challenges, including how to further improve targeting, biocompatibility, and immunogenicity, as well as how to overcome biological barriers and improve the stability of drug delivery [110]. Future studies need to focus on optimizing the design of nanocarriers to improve the safety and efficacy of their clinical applications while reducing potential side effects. As nanotechnology continues to advance, it is expected that these challenges will be gradually overcome and nano-DDSs will play an increasingly important role in the treatment of CVDs. In terms of clinical treatment, research on nanomaterial delivery has moved from the laboratory to clinical trials [111]. Several clinical trials have been registered to explore the use of these nanocarriers in the diagnosis and treatment of CVDs [112]. The success of these trials will provide more effective and safer treatment options for patients with CVDs, ultimately improving patient care and outcomes. Future studies should continue to explore the potential of these nanocarriers in clinical therapeutics, particularly in terms of improving the precision of drug delivery, reducing systemic side effects, and promoting the repair and regeneration of damaged cardiac tissue. As we gain a better understanding of these nanosystems and progress in clinical trials, we can hope to achieve more personalized and precise treatment options for cardiovascular disease in the future.

## Figures and Tables

**Table 1:** Characteristic differences in viral vectors.

Characterization	LV	AdV	AAV
Genome Type	RNA	dsDNA	ssDNA
Host Genome Integration	Integration	Non-integration	Non-integration
Duration of Expression	Long	Short or medium	Medium or long
Immunogenicity	Low to medium	Medium to high	Low
Gene Carrying Capacity	Larger (up to 9 kb)	Larger (up to 7.5–8 kb)	Smaller (5 kb)
Dominance	Long-term stable expression	High-efficiency transfection; broad tissue tropism; vaccine development	Low immunogenicity; High safety; cardiac affinity
Challenge	Mutations	Immune response; transient gene expression	Limited carrying capacity; pre-existing immunity; high cost
Applications	Cardiac transplantation	Myocardial perfusion; angina symptoms	Myocardial infarction, Myocardial hypertrophy and atherosclerosis
References	[49]	[53]	[55,56,57,58,59,60]

## Data Availability

No data are associated in the manuscript.
